# Biosynthesis and Pharmacological Activities of the Bioactive Compounds of White Mulberry (*Morus alba*): Current Paradigms and Future Challenges

**DOI:** 10.3390/biology13070506

**Published:** 2024-07-07

**Authors:** Maryam Fatima, Mudasir A. Dar, Maruti J. Dhanavade, Syed Zaghum Abbas, Mohd Nadeem Bukhari, Abdullah Arsalan, Yangzhen Liao, Jingqiong Wan, Jehangir Shah Syed Bukhari, Zhen Ouyang

**Affiliations:** 1 School of Pharmacy, Jiangsu University, Zhenjiang 212013, China; 1000006498@ujs.edu.cn (M.F.);; 2 School of the Environment and Safety Engineering, Biofuel Institute, Jiangsu University, Zhenjiang 212013, China; muddar7@ujs.edu.cn; 3 Department of Microbiology, Bharati Vidyapeeth’s Dr Patangrao Kadam Mahavidyalaya, Sangli 416416, India; 4 College of Engineering, Institute of Energy Infrastructure, Universiti Tenaga Nasional, Jalan Kajang-Puchong, Kajang 43000, Selangor, Malaysia; 5 School of Textile and Clothing, Qingdao University, Qingdao 266071, China; 6 School of Food and Biological Engineering, Jiangsu University, Zhenjiang 212013, China; 7 School of Chemistry and Chemical Engineering, Hunan University, Changsha 410082, China

**Keywords:** *Morus alba*, glycoside flavonoids, pharmacology, biological activities, phytochemical composition, bioactive compounds, biosynthesis

## Abstract

**Simple Summary:**

The presence of secondary metabolites like flavonoids, alkaloids, and phenolic compounds in white mulberry (*Morus alba*) make it an ornamental tree mainly in the Asian subcontinent. These secondary metabolites are synthesized through various biosynthetic pathways. Flavonoids like rutin, quercetin, and kaempferol are produced via the phenylpropanoid and flavonoid biosynthetic pathways. The key enzymes responsible for this pathway include phenylalanine ammonia-lyase, chalcone synthase, and flavonoid hydroxylases. Phenolic compounds like resveratrol and oxyresveratrol are produced via the phenylpropanoid pathway using phenylalanine as a precursor. Presences of these secondary metabolites, like in the case of flavonoids (rutin and quercetin), in the mulberry tree have potential biological activities like antioxidant, anti-inflammatory and neuroprotective effects. Also, they have been found to have great medicinal importance, for example in treating diabetes, obesity and neurodegenerative diseases. Also, phenolic compounds like reservertrol exhibit cardio-protective, anticancer, and anti-aging properties.

**Abstract:**

Traditional natural products have been the focus of research to explore their medicinal properties. One such medicinally important plant is the white mulberry, *Morus alba*, widely distributed in the Asian subcontinent. It is one of the most cultivated species of mulberry tree and has attracted more focus from researchers because of its abundance in phytochemicals as well as multipurpose uses. The leaves, fruits and other parts of the white mulberry plant act as a source of valuable bioactive compounds like flavonoids, phenolic acids, terpenoids and alkaloids. These secondary metabolites have manifold healthy uses as they possess antioxidant, anti-inflammatory, antidiabetic, neutrotrophic, and anticancer properties. Despite the increasing scientific interest in this plant, there are very few reviews that highlight the phytochemistry and biological potential of white mulberry for biomedical research. To this end, this review elaborates the phytochemistry, biosynthetic pathways and pharmacological activities of the glycoside flavonoids of *Morus alba*. A comprehensive analysis of the available literature indicates that *Morus alba* could emerge as a promising natural agent to combat diverse conditions including diabetes, cancer, inflammation and infectious diseases. To achieve such important objectives, it is crucial to elucidate the biosynthesis and regulation mechanisms of the bioactive compounds in white mulberry as well as the multifaceted pharmacological effects attributed to this plant resource. The present review paper is intended to present a summary of existing scientific data and a guide for further research in the phytochemistry and pharmacology of white mulberry. Further, a biosynthetic pathway analysis of the glycoside flavonoid in mulberry is also given. Lastly, we discuss the pros and cons of the current research to ensure the prudent and effective therapeutic value of mulberry for promoting human and animal health.

## 1. Introduction

Organic products present in animals, plants and minerals plays a important role in the field of medicinal chemistry; therefore, researchers are focused on the extraction, isolation, identification, and characterization of these organic products [1]. Conventional medicines use phytochemical-rich plant extracts in the treatment of many diseases because natural products are effective, although they may have numerous side effects [2]. This makes plants one of the best resources for the discovery of new compounds that serve as precursors for drug development [3]. Medicinal plants of the Moraceae family are known for their versatile applications in many fields including cosmetics, agriculture and pharmaceuticals. One example of a Morus plant is *Morus alba*, also known as white mulberry. Being indigenous to China, mulberry grows throughout the world due to sericulture practices [4]. Historically, all parts of the mulberry tree have been used to address a spectrum of physiological conditions, including cooling, sedation, diuresis, tonicity, neuropathy,, etc. [3]. In particular, its leaves have been employed as sweat inducers, cooling agents, and antipyretic agents, in addition to their nutritional value as rich sources of proteins and polysaccharides [5]. The primary bioactive compounds that are present in mulberry leaves include flavonoids and amino acids, polysaccharides, vitamins, and steroids that can be used to treat various internal diseases and infections. Among them, flavonoids have emerged as a major component for research over the past few years due to their potential biological properties, like anti-inflammatory, anti-aging, and anti-hyperglycemic activities [5,6]. Flavonoids are a large group containing at least 6000 molecules which are classified into six categories: aurones, isoflavonoids, flavones, phlobaphenes, anthocyanins and flavonols [7]. The flavonoid compounds commonly found in mulberry include kuwanon (flavons), sangenon (flavanols), rutin (flavons), quercetin (flavonols) and catechins (flavanols). Flavonoids are mostly found in glycosylated form in plants and glycosylation moderates flavonoids by increasing their solubility and stability. They play a pivotal role in plant metabolism. Polyphenolic flavonoids, benzofurans, stilbenes, and Diels–Alder adducts, are the primary chemical constituents with bioactive potentials within Morus plants. These polyphenols derived from Morus plants demonstrate diverse bioactivities, including anti-oxidative, antidiabetic, cytotoxic, antimicrobial, anti-inflammatory, and skin-whitening effects [8]. Notably, the antidiabetic efficacy has been substantiated recently through various clinical studies [4].

Compared to *Morus rubra* (red mulberry) and *Morus nigra* (black mulberry), *Morus alba* (white mulberry) has garnered greater attention from researchers for a number of important reasons. Although *Morus alba* is indigenous to China, it has been widely farmed and allowed to naturally occur over much of the world, including Asia, Europe, and North America. *Morus alba* is now more widely available for research, with a greater pool of plant material and research samples thanks to its broad dissemination and cultivation. The relatively restricted natural ranges of *Morus rubra* and *Morus nigra*, on the other hand, may limit the availability of study resources. Because of its significance in the global sericulture (silk production) business, *Morus alba* has a substantial economic impact.

*Morus alba* is now more widely available for research, with a greater pool of plant material and research samples thanks to its broad dissemination and cultivation. The relatively restricted natural ranges of *Morus rubra* and *Morus nigra*, on the other hand, may limit the availability of study resources. Because of its significance in the global sericulture (silk production) business, *Morus alba* has a substantial economic impact. The leaves of *Morus alba* are the primary food source for silkworms, making it a crucial crop in many silk-producing countries [9]. Extensive research has been conducted to optimize the usage of *Morus alba* in the silk industry by examining its biological features, composition, and growing methods, due to its economic relevance. For its numerous bioactive components, pharmacological actions and possible health advantages, *Morus alba* has been the subject of in-depth research. There is a substantial body of research literature available on *Morus alba*, providing a solid foundation for further investigations [10,11]. In comparison, the research on *Morus rubra* and *Morus nigra*, while developing, is considerably less extensive, forcing researchers to build a fresh knowledge base. *Morus alba* is known for its ability to adapt to a wide range of climatic conditions and soil types, making it easier to cultivate and maintain for research purposes [12]. This adaptability and hardiness can simplify the logistics and consistency of research involving *Morus alba*, compared to the more specialized requirements of *Morus rubra* and *Morus nigra*. *Morus alba* has been found to contain a rich array of bioactive compounds, including polyphenols, flavonoids and other phytochemicals [9,11]. These bioactive compounds have been associated with various health-promoting effects, such as antioxidant, anti-inflammatory, antidiabetic, and neuroprotective properties. The potential therapeutic applications of *Morus alba* have generated significant research interest in exploring its biological activities and mechanisms of action. While *Morus rubra* and *Morus nigra* have their own unique bioactive profiles and potential benefits, the broader availability, economic importance, established research history and adaptability of *Morus alba* have contributed to its greater research interest and focus compared to the other two mulberry species.

Few reviews have been published about the pharmacotherapeutics of *Morus alba* [2] and the comprehensive details of the bioactive compounds extracted from its leaves and their potential biomedical applications are still largely lacking. Therefore, this review presents a comprehensive account of the biological and chemical compounds found in the leaves of *Morus alba*. Further scope of this review aims to discover novel phyto constituents of *Morus alba* and their pharmaceutical exploration through advanced research techniques. The presences of rich secondary metabolites like flavonoids, alkaloids and phenolic compounds in white mulberry (*Morus alba*) make it an ornamental tree due to their medicinal importance. Due to these secondary metabolites in mulberry trees, they possess potential biological activities like antioxidant, anti-inflammatory and neuroprotective effects. Also, they have been found to have great medicinal importance, for example in treating diabetes, obesity and neurodegenerative diseases. Additionally phenolic compounds like resveratrol exhibit cardio-protective, anticancer, and anti-aging properties. Owing to these important benefits of the trees, we herein succinctly summarize the promising properties exhibited by the flavonoid contents of mulberry. The insights derived from this review hold potential benefits for the development of nutritional supplements and highlight the potential of *Morus alba* extracts as prospective therapeutics in the future. Further, this review aims to discover novel phytoconstituents of *Morus alba* and their pharmaceutical exploration through advanced research techniques.

## 2. Methodology

**Figure 1:**
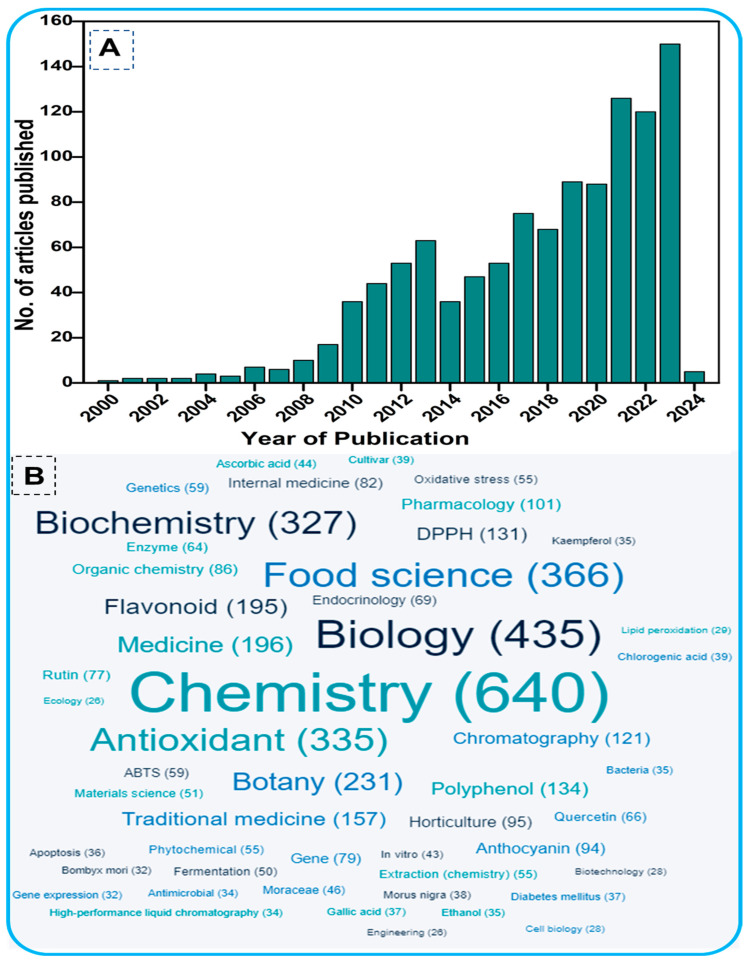
(**A**). Total number of articles published from 2000 onwards on flavonoids of mulberry trees. The data were retrieved from the PubMed and Scopus databases using the keywords Mulberry AND Flavonoids AND Phytochemistry in the title/abstract/keywords of published papers. (**B**). Word cloud of the interdisciplinary fields within the research topic.

For this study, a comprehensive literature search was performed in the PubMed (https://pubmed.ncbi.nlm.nih.gov/ (accessed on 11 April 2024)) and Scopus databases (http://www.scopus.com/ (accessed on 11 April 2024)). These databases are well-known data resources and are preferred for bibliometric analysis. The terminology used included “Mulberry” plus “Flavonoids” plus “Phytochemistry” as key terms. The search period ranged from the year 2000 onwards (Figure 1), restricted to research papers only. The total number of relevant papers was 1107 with more than 24,000 citations. Figure 1 shows the evolution of the subject over the past two decades, based on the number of scholarly works published, highlighting the tremendous attention given by the scientific community to the research topic. Figure 1B shows the word cloud analysis of the phytochemicals of mulberry leaves, with chemistry being the dominant subject. The identified subject domains indicate that the studies are mainly related to the chemistry and phytochemical properties, particularly antioxidant activity, of mulberry plants.

**Figure 2:**
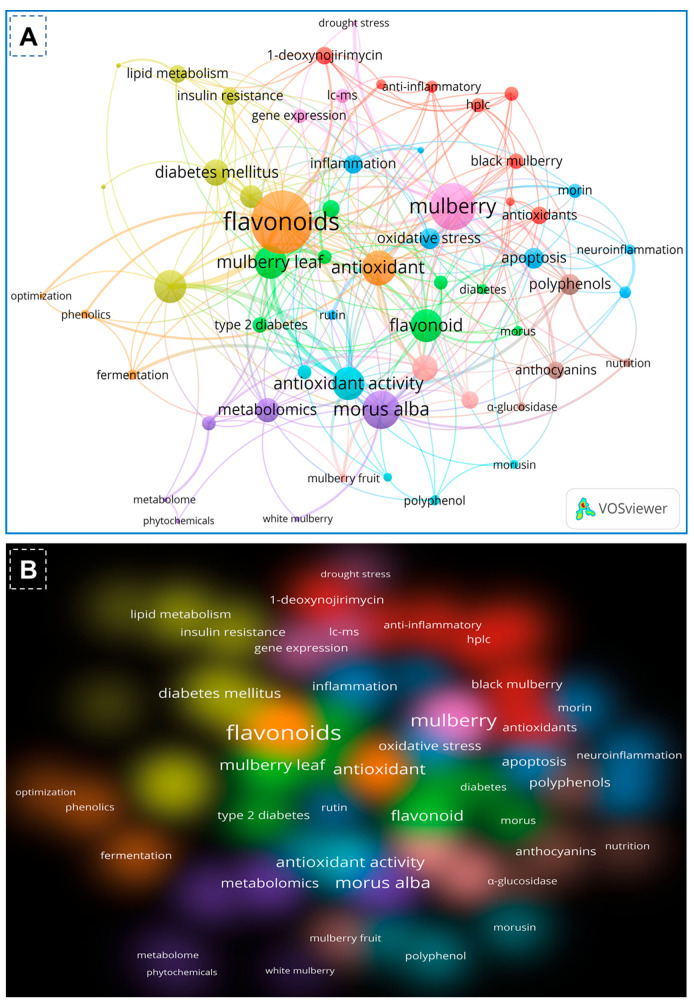
Co-occurrence network analysis of author keywords with a word frequency of more than five times. The data were obtained from a literature search with flavonoids of mulberry leaves using the full counting method. (**A**) The collaboration analysis of flavonoids and mulberry. (**B**) The density visualization of flavonoids of mulberry and their applications depicted by the full counting method.

On the basis of the PubMed and Scopus databases, a VOSviewer version 1.6.20 for bibliometric analysis of flavonoids of mulberry studies has been developed. According to the findings of the VOS investigation, the body of knowledge surrounding flavonoids of mulberry has expanded significantly, with current research concentrating primarily on phytochemistry of mulberry and its biological activities including antioxidant potentials. By examining the co-occurrence of author keywords in the literature, Figure 2A demonstrated the collaborative analysis of flavonoids and mulberry. The minimum number of keyword occurrences required to meet 58 of the 1482 keywords was four. In the VOS viewer, the 58 keywords were divided into ten clusters, with the first three clusters comprising 8 keywords, fourth cluster comprising 7 items, and the fifth cluster having 6 items. Each cluster was denoted by a distinct color to illustrate the relationship between its constituents. Clusters of identical terms typically have stronger relationships. The first cluster demonstrated the link between mulberry and antioxidant and anti-inflammatory effects, 1-deoxynorjirimycin, resveratrol, and quercetin. The second cluster is characterized by essential terms including flavonoids, *Morus*, mulberry leaf, moraceae, diabetes, pharmacology and molecular docking. The third cluster, however, demonstrated the relationship between mulberry and its uses as indicated by key terms such as morin, herbal medicine, Alzheimer’s disease, apoptosis, inflammation, neuroinflammation, oxidative stress and rutin. Keywords such as *Diabetes milletus*, gut microbiota, insulin resistance, lipid metabolism, mulberry leaves and traditional Chinese medicine were used to identify the antidiabetic applications of mulberry leaves in cluster four. The keywords anthocyanin, nutrition, polyphenols and α-glucosidases within cluster four further revealed the bioactive functions of mulberry flavonoids. Similarly, the remaining clusters demonstrated advanced technologies like metabolomic, transcriptomics, gene expression, fermentation and drought stress of the mulberry. In recent years, biomedical applications of mulberry phytochemicals particularly flavonoids, towards diabetes, lipid metabolism and nutrition has attracted a significant amount of interest. Figure 2B depicts the density visualization of flavonoids from *Morus alba* with *diabetes mellitus* and antioxidant/antioxidant activity, which reveals a clear link between mulberry flavonoids and pharmacological applications.

## 3. Phytochemistry and Nutritional Values of Mulberry Leaves

**Figure 3:**
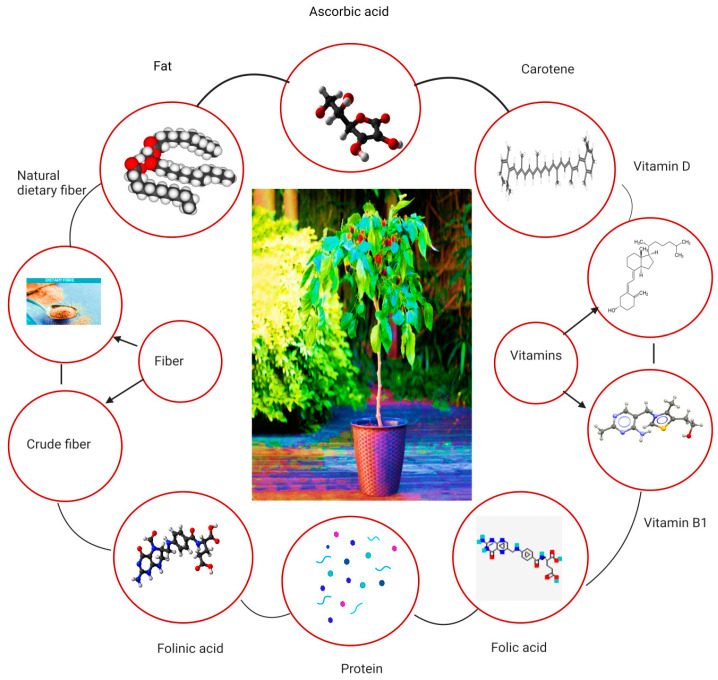
An overview of the nutritional content of the mulberry leaves.

The nutritional composition of *Morus alba* leaves is extensive, encompassing various macronutrients, micronutrients and bioactive compounds. Amino acids, phenols, polysaccharides, steroids, lignin and volatile components are the main chemical compositions present in the leaves of mulberry. Ascorbic acid is present in mulberry leaves. Moreover, vitamin B1, folic acid, carotene folinic acid and vitamin D are also present in mulberry leaves. In dry conditions, the nutritional values of mulberry leaves have 15–31% protein, 2–8% fat, 10–14% crude fiber, 27–43% neutral dietary fiber (NDF) and 11–17% ash [13]. It has also been found that mulberry leaves contain other therapeutic components such as Moran 20k and 1-deoxynojirimycin (DNJ) as antidiabetic agents and isoprene-substituted flavanones such as Kuwanon C and kuwanon as antimicrobial agents [14]. Figure 3 depicts the overall nutritional aspects of mulberry leaves. A thorough analysis of the available literature underscores the significant variability in both the quantity and quality of the bioactive and nutritional compounds present in mulberry leaves, with distinctions evident across different cultivars [15]. For instance, the DNJ content of the Japan cultivar *Morus alba* Ichinose was relatively low (53 mg/100 g DW) when compared to the quantity of the same compound in the Spain cultivar containing 213 mg/100 g DW [16,17]. The overall amount of DNJ in different cultivars of *Morus alba* has been detected within a range of 0.103% to 0.2% [18]. Similarly, notable differences in the contents of crude protein (13.4–24.36%), crude fat (4.24–8.02%), total carbohydrate (47.27–56.42%), potassium (1.2 g/100 g–3.9 g/100 g), calcium (1.7 g/100 g–3.9 g/100 g) and iron (119.3 mg/kg–241.8 mg/kg) have been observed among different cultivars of *Morus alba* cultivated across the globe [3]. The organic acid contents such as citric acid, malic acid and ascorbic acid extracted from the leaves of different cultivars of *Morus alba* range between 32.2 and 105.5 mg/100 g, 43.7 and 72.6 mg/100 g and 0.97 and 1.49 mg/g respectively. Furthermore, the total phenolic and flavonoid concentrations of *M. alba* leaves span a range from 0.95 to 2.39 mg/g DW and 2.64 to 7.33 mg/g DW, respectively, in addition to containing 20.05% alkaloids [19,20,21,22,23,24,25,26,27,28,29]. The variations in the phytochemistry and nutritional contents could be attributed to the differences in geographical and environmental factors including climate, soil composition, altitude, sunlight and precipitation,, etc. [13,20]. In this context, there is a critical need for comprehensive analyses focused on genomic, metabolomic and proteomic approaches to pinpoint the pivotal genes and metabolic pathways influenced by geographical and environmental factors within distinct cultivars. Such investigations are indispensable to elucidate the complex genetic and biochemical mechanisms underlying the discernible variations in bioactive and nutritional compounds observed across different cultivars.

### 3.1. Flavonoids

**Figure 4:**
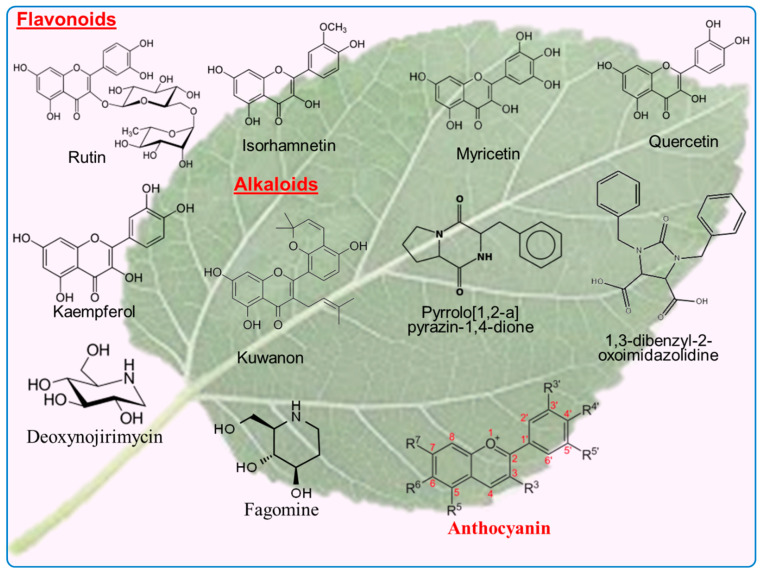
The chemical structure of flavonoids and alkaloids present in the leaves of mulberry tree.

The list of the flavonoid compounds extracted from mulberry plants using different methods is shown in Table 1. The presence of abundant flavonoids in Morus plants are well known (Figure 4). Quercetin, kaempferol, Kuwanons, moracin flavans, moragols and morkkotins are well known flavonoids in mulberry leaves [30]. Rutin, kaempferol and astragalin are found in the range from 4.34 mg/g to 0.53 mg/g of all total content [31]. Glycosides, flavonol, quercitin-3-(6-malonylglucoside), rutin, quercitin-3-(6-malonylglucoside) and isoquercitin, which possess strong antioxidant potential, are extracted from the leaves of mulberry [5]. As well as having nutritional values, flavonoids are highly useful as bioactive compounds against many ailments, including allergic and viral infections [32].

### 3.2. Alkaloids

1-deoxynojirimycin (1-DNJ), 4-O-β-D-dlucopyranosyl-fagomine (Glu-FAG), D-fagomine (FAG), 2-O-α, D, aurantiamide acetate, galactopyranosyl-deoxynojirimycin (GAL-DNJ), isofagomine, cis-5-hydroxypipecolic acid, trans-5-hydroxypipecolic acid, methylpyrrolidine carboxylic acid and pipecolic acid are the main alkaloids that are found in high concentrations in mulberry leaves. The active component of mulberry leaves is 1-deoxynojirimycin (1-DNJ, which has been given a lot of attention by researchers because of its effective and specific inhibition of various enzymes which take part in various vital biochemical processes such as maturation of sugar chains in glycoproteins and intestinal digestion [49]. DNJ is an anti-hyperglycemic agent that slows the rate of carbohydrate breakdown to monosaccharides, and it can also inhibit glucose absorption and post-prandial blood glucose levels [50]. In mulberry leaves, a total of 21 alkaloids have been identified and isolated.

### 3.3. Anthocyanins

Anthocyanins, natural phenolic compounds, are responsible for the colors of fruits, flowers and leaves. While mulberry leaves are not typically known for their high anthocyanin content, certain varieties of mulberry trees may contain small amounts of these compounds [51]. Anthocyanins are more commonly found in mulberry fruits rather than in the leaves. However, mulberry leaves offer other nutritional benefits, including vitamins, minerals and other phytonutrients, making them a valuable component in traditional medicine and culinary practices. Anthocyanin production in plants is achieved by the manufacture of phenylpropanoids, which is assisted by a series of enzymatic processes mediated by enzymes such as 4-coumarate-CoA ligase, cinnamate 4-hydroxylase and phenylalanine ammonia lyase. The production of anthocyanin is controlled by transcription factors such as Myeloblastosis transcription factor, basic helix-loop-helix (bHLH) transcription factor and WD40-repeat proteins. Moreover, a variety of stages in the process of anthocyanin production are controlled by the R2R3- Myeloblastosis transcription factor [52]. Anthocyanins are the best source for health benefits as they act as antioxidants, anti-inflammatories, etc. Anthocyanins have very high inhibitory ability on lipid oxidation and have anti-metastasis activity to inhibit migration of B16-F1 cells [53,54].

### 3.4. Polysaccharides

The main bioactive components present in mulberry leaves are polysaccharides. They are long-chain macromolecular polymers that are generally used as thickeners and gelling agents in the food industry because of their ability to retain water and form hydrogels. Due to the rich biological activities of polysaccharides in mulberry leaves, they have been receiving increasing attention from the scientific community. These polysaccharides promote insulin expression and regulate liver glucose metabolism [55]. In mulberry leaves, polysaccharides are mainly present in the inner epidermal cells [56]. Galactose, glucose, arabinose, xylose, fructose, rhamnose, galacturonic acid, glucuronic acid mannose and sorbose are monosaccharides that combine with each other and form many types of polysaccharides in mulberry leaves [57].

### 3.5. Amino Acids

Amino acids are the building blocks for the synthesis of proteins, which includes antioxidant enzymes. Some of the amino acids can directly scavenge oxygen free radicals. Among the amino acids, the four that are mainly present in mulberry leaves, include Asparagine, Alanine, Proline and GABA. Mulberry leaves contain high amino acid contents which are nutritively superior and also promote the growth and development of silkworms [58].

## 4. Biosynthetic Pathway for Flavonoids

**Figure 5:**
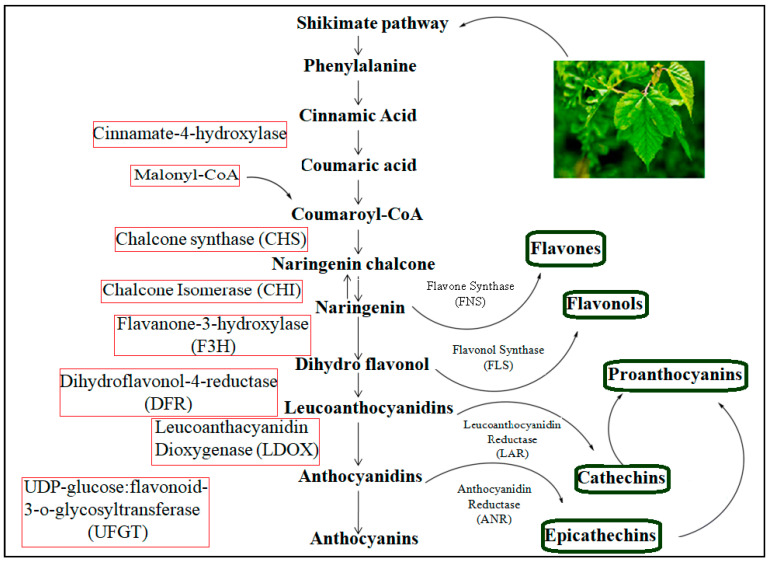
Phenylpropanoid pathway for flavonoids.

For producing structurally diverse natural products, the more efficient and greener pathway is the biosynthetic pathway. Biosynthesis can be used in simple and complex transformations without disturbing the blocking and deblocking steps, which are common in organic synthesis [59]. However, a problem regarding the biosynthetic pathway route in mulberry leaves is in understanding the mechanism, which is not clear, and currently, there is striking emphasis from scientists to understand the molecular mechanism of flavonoids. The shikimate pathway is followed by the phenylpropanoid metabolic pathway and possesses approximately 15 carbon atoms arranged in three aromatic rings linked as C6-C3-C6. It is used for flavonoid synthesis [60]. There are several enzymes that are involved in the shikimate pathway, which is a six-step reaction in the biosynthesis of shikimic acid. The reaction, as shown in Figure 5, begins with a simple aldol condensation reaction of phosphoenol pyruvic acid and D-erythrose 4-phosphate [61]. The end product of chorismic acid is converted into the amino acid phenylalanine by the action of prephenate-aminotransferase (PhAT) and aromate-dehydrate (ADT) enzymes [62].

Flavonoid biosynthesis begins with the condensing of one p-coumaroyl-CoA molecule (shikimate-derived, B ring) with three molecules of malonyl-CoA (polyketid origin, A ring) to give chalcone (2′4′6′4-tetrahydroxychalcone) catalyzed by chalcone synthase (CHS). Chalcones are then converted to flavanones by chalcone flavanone isomerase (CHI). From these central intermediates, the pathway branches into several side branches that generate another type of flavonoids. The initial part of these pathways is known as the general phenylpropanoid pathway [18,63]. In the phenylpropanoid pathway, an aromatic amino acid, phenylalanine, is first converted to p-coumaroyl-CoA through the activity of phenylalanine ammonia lyase (PAL), cinnamic acid 4-hydroxylase (C_4_H) and 4-coumarate-CoA ligase (4CL). The PAL catalyzes the deamination of phenylalanine to trans-cinnamic acid in the first committed step, referred to as the deamination of phenylalanine to trans-cinnamic acid. The second step of the phenylpropanoid pathway is enzymatically mediated C_4_H, a cytochrome P450 monooxygenase, in plants. This enzyme is responsible for the conversion of trans-cinnamic acid to p-coumaric acid through hydroxylation. The third step in the general phenylpropanoid pathway involves the activation of p-coumaroyl-CoA by the action of 4-courmarate: CoA ligase. In mulberry plants, the activity of 4CL is positively correlated with anthocyanin and flavonol content in response to stress [64], whereas PAL, C_4_H and 4CL are generally coordinately expressed. Chalcone synthase (CHS) is involved in the successive acetylation of three acetate moieties to produce naringenin chalcones. In vitro, it has been shown that naringenin calcone is converted to naringenin through chalcone isomerase (CHI) [65]. Naringenin is a very significant flavonoid frame that is converted in the presence of FNS I and FNS II (flavone synthases I and II) and IFS (isoflavone synthase) to flavones and isoflavones, respectively [66]. Naringen can also be catalyzed by flavanone-3-hydroxylase (F3H), flavonol-3′-hydroxylase (F3′H) and flavonol-3′5′-hydroxylase (F3′5′H) to synthesize dihydro-myricetin, dihydro-kaempferol and dihydro-quercetin, respectively [67]. The flavonol synthase (FLS) converts dihydro flavonols into flavonols (like kaempferol, quercetin and myricetin), which are further catalyzed by dihydroflavonol 4-reductase (DFR) to generate leucoanthocyanidins [68]. Leucoanthocyanidins are oxidized by leucoanthocyanidin dioxygenase (LDOX) to produce anthocyanidins [69]. Next, the anthocyanidins and leucoanthcyanidins are converted into proanthocyanidins through the action of leucoanthocyanidin reductase (LAR) and anthocyanidien reducatse (ANR) in a sequential manner [70]. The additional reactions, that include glycosylation, methylation and acylation, help to enhance the stability of the vacuolar anthocyanins [71]. The phenylpropanoid pathway of the flavonoids in mulberry is represented below in Figure 5. The metabolic pathway continues through a series of enzymatic modifications to yield flavonols, dihydroflavonols and anthocyanins.

### 4.1. Related Functional Genes in the Biosynthetic Pathway of Flavonoids in Mulberry

The regulation of the gene pairs is crucial for the proper biosynthesis of the flavonoid pathways. Huang Manfen cloned some of the genes by using the cDNA of immature mulberry leaves of the *Morus multicaulis* variety 71-1 as a template. The full length sequences for the cinnamic acid-4-hydroxylase (MmC4H, GenBank entry number: KJ013408), 4-Coumaric acid-CoA ligase (Mm4CL, GenBank accession number: KJ013409) and Chalcone synthase (MmCHS, GenBank entry number: KJ013407) genes are already available. Furthermore, there is a correlation between the total flavonoid content of mulberry leaves and the expression levels of the three associated genes [72].

Zhao et al. (2015) used *Morus alba* L. transcriptome sequencing to examine 21 linked genes in the phenylpropanoid pathway [73]. The authors studied the expression and accumulation of rutin in various tissues of the mulberry plant. The accumulation of rutin in mulberry leaves was 5 times higher than their wild counterparts. The MaUGT78D1 gene may be crucial to the rutin biosynthesis pathway as per the connotation found between the expression levels of associated genes and the accumulation of rutin. The accumulation of polyphenols in mulberry shows variations; however, Wei and colleagues (2017) studied PAL and F3H in mulberry leaves and showed a certain degree of positive correlation with the accumulation of polyphenols [74]. The authors also cloned and acquired the CHS gene in 2017 using *Morus alba* L. using cDNA as a template. It was also discovered that the MaCHS fragment was 515 bp long and that it had a 345 bp open reading frame (ORF) region that encodes 115 amino acids. The isoelectronic point of the acid is 5.43 and the calculated molecular mass is 13,202.2 [75]. Many recent studies have shown that PAL, C4H, 4CL, CHS, F3H, F3’H and DFR are the main enzyme genes involved in the present biosynthesis route of flavonoids [76]. While cloning these genes has been a focus, there has been comparatively little research on their functions.

### 4.2. Factors Effecting the Biosynthesis of Flavonoids in Mulberry

The expression of the genes encoding for flavonoids in the mulberry is influenced by several factors, including both physical (light, water availability and temperature) and physiological parameters, such as hormones (like jasmonic acid) and injuries [77].

#### 4.2.1. Effect of Temperature on Flavonoid Biosynthesis

Temperature is an important environmental factor that influences the biosynthesis of flavonoids in plants. Many studies have reported that a low temperature is more favorable for the accumulation of flavonoids in mulberry. For example, Caldwell et al. studied the effect of rising temperature on the isoflavone content in soybeans and found that isoflavone content decreased by 65% when rising from 18 °C to 23 °C and by approximately 90% when rising to 28 °C [78].

There are three main reasons for the increase in flavonoid content under low temperature conditions:

(a) The activity of the related enzymes increases at low temperatures in flavonoid biosynthesis pathways. Boo et al. found that the content of total polyphenols and anthocyanins was highest at low temperatures and the activity of phenylalanine ammonia-lyase was also too high at low temperatures [79]. (b) At the low temperature, the gene expression also increases. Crifò et al. investigated the expression levels of anthocyanins-related genes in mulberry fruits of two cultivars and four samples and their effects on anthocyanin biosynthesis. There was a significant increase in the transcript levels of all genes which were studied (CM1, PAL, CHS, DFR, ANS, UFGT and GST) [80]. (c) As the temperature difference between day and night increases, this can promote the biosynthesis of flavonoids. Cohen et al. studied the effects of day and night temperature changes on fruit growth, proanthocyanidine accumulation and flavonoid biosynthesis genes. Among the expression effects, it was found that berry warming and cooling altered the initial rate of proanthocyanidin accumulation, which was related to the flavonoid synthesis pathway. The expression of the nuclear genes is significantly correlated [81].

#### 4.2.2. Effect of Light

The most important environmental factor influencing the biosynthesis pathway of flavonoids in plants is light. The rapid induction of flavonoid biosynthesis is generally observed under strong light conditions, which reflects the important role of flavonoids in photoprotection. However, in some fruit species, flavonoid biosynthesis is less influenced by light. The dependence of light on plant development has driven the evolution of some sophisticated mechanisms for sensing different aspects of light signals. The effect of light can further be categorized into photoperiod (duration), intensity (quantity), direction and quality (wavelength), including UV radiations. Further, the duration of exposure to the light also determines the accumulation of the flavonoid contents in different varieties of mulberry like Shiraz [82], Pinot Noir [83] and Sangiovese [84]. The expression of some flavonoid genes was reduced by shading treatment [82]. In particular, the influence of light quality was investigated [85]. Covering the plants with a UV-resistant film has shown no effect on the amount of proanthocynidins, but this treatment significantly reduces the flavonols [86].

#### 4.2.3. Effect of Water Availability

The changes in the soil water content affect the synthesis of secondary metabolites in medicinal plants by the variations in gene transcription. The affected genes in turn can respond synergistically to changes in soil water and this may alter the flavonoid concentration in leaves and roots. Grimplet and coworkers found that water deprivation causes upregulation of mRNA involved in various pathways of secondary metabolism. This phenomenon is mainly limited to the pulp and skin tissues. The seeds are rarely affected by this phenomenon [87]. A deficiency of water also induces an increase in the expression of the grape BTL homolog, in parallel with the known macroscopic effects on berry pigmentation [88] and also the activation of the flavonoid biosynthesis pathway [89].

## 5. Pharmacological Activities of Mulberry Leaves

**Figure 6:**
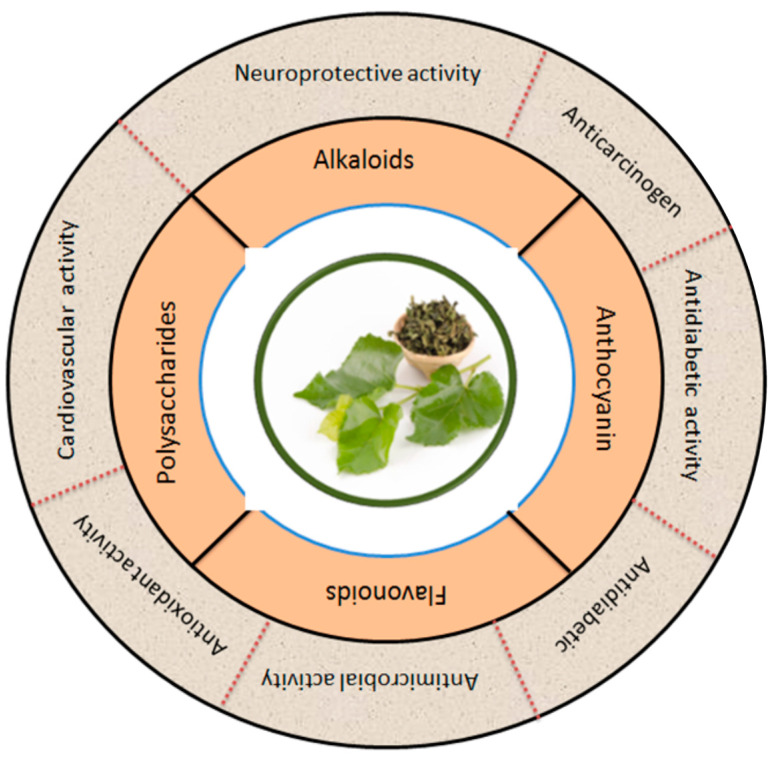
Mulberry leaves are a natural source of many bioactive compounds, which have been increasingly recognized for their potential to promote human health. The figure shows the pharmacological applications of bioactive compounds extracted from mulberry leaves. The figure also illustrates the various bioactive compounds found in mulberry leaves and their corresponding potential health applications. Modified from [18,21,92,93].

Figure 6 summarizes the phytochemicals and biological activity of mulberry leaves in broad categories. Apart from sericulture, mulberry leaves are also used as medicine in most Asian countries, especially in China [90]. The major bioactive ingredients in plants are flavonoids. Mulberry leaves are rich in flavonoids and many other bioactive compounds which function to lower the blood sugars, reduce blood lipids, resist viruses, slow aging and enhance immunity [91]. The phenolic compounds in flavonoids enable them to work as metal chelators and antioxidants. Flavonoids may also act as potential anticancer promoters and cancer chemopreventive agents [3]. An overview of the different activities of the bioactive compounds extracted from the leaves of the mulberry is shown in Table 2. 

### 5.1. Antioxidant Effects of Mulberry Leaves

**Figure 7:**
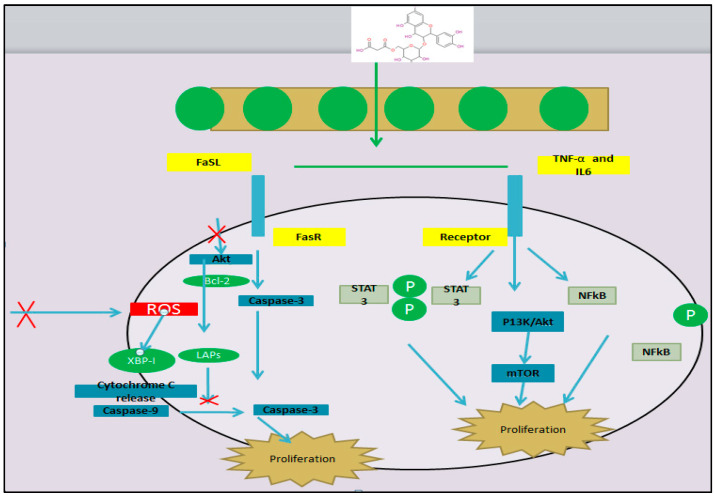
Mechanism of mulberry leaves as anticancer agents.

The mechanism of antioxidants is shown in Figure 7. Some of the withstanding agents help to prevent deterioration by oxidation, for instance fats, oils, foods,, etc., and certain compounds like vitamin C and vitamin E coalesce to counter the detrimental impact of oxidation to the lives of the living organisms. The free radicals possess unpaired electrons, which are considered as fragments of molecules that are generally very reactive. They are continuously produced in the cells as by-products of metabolism [94]. It is then important to note that plants have the inherent ability to produce some particular protective anti-oxidative defense enzymes such as catalases, peroxidases, polyphenol oxidases, ascorbate peroxidases and glutathione reductases under any given environmental stress [95]. To avoid threats related to oxidative stress, antioxidants are required in the form of food supplements [96].

The presence of different types of flavonoids in mulberry leaves helps it to be a good antioxidant. The increased antioxidative capability of mulberry leaves can be correlated with higher contents of phenols and flavonoids [23,24]. Flavonoids with high antioxidant activity can be used in many ways; for example, (i) they can inhibit reactive oxygen species inhibition and (ii) leukocyte immobilization, and (iii) inhibit nitric oxide and (iv) xanthine oxidase inhibition [97]. Since these flavonoids have physiological functions in plants, they are also essential for human consumption [98]. This is perhaps due to the higher concentration of quercetin in mulberry leaves that perhaps slows the rate of oxidation [6]. Three flavonoids, quercetin, rutin and isoquercetin, are considered as main antioxidant components present in the ethanol extracts of mulberry leaves [5].

### 5.2. Anti-Carcinogenesis by Mulberry Leaf Extracts

Flavonoids have been ranked as being effective in cancer prevention and some selected isoflaones are known to suppress cancer in several animal models [99]. Some studies revealed that flavonoids inhibit carcinogenesis in vitro and significant results also indicate that they also achieve this in vivo. Among the phenolic compounds, flavonoids are the most abundant in mulberry leaves [15,100,101]. The results of the present study also reveal that the extracts of mulberry leaves possess positive cytotoxic effects against cancerous cells. The galactose-bonded lectin isolated from mulberry leaves has cytotoxic activity on human breast cancer with IC_50_-8.5 μg/mL and also on colon cancer cells with IC_50_-16 μg/mL [102]. Many phenolic compounds are present in mulberry leaves, which induce anticancer activity in hepatoma cells by capturing the cell cycle at the G2-M phase and inhibit topo-isomerase II activity. Anthocyanins are also a phenolic compound present in mulberry leaves and have beneficial effects by decreasing the risk of cancer because of their high chemopreventive properties [103]. Another flavonoid present in mulberry leaves is quercetin, which is capable of reducing the progress of human β leukemia [104]. Many other clinical trials revealed the therapeutic potential of mulberry leaves against cytotoxicity as a cheap and easily available source for the treatment of cancer which reduces the invasiveness of cancer cells. The flavonoids and quercetins present in mulberry leaves also exhibited selective cytotoxicity against human ovarian cancer and gastric cancer [105].

### 5.3. Antidiabetic Activity

**Figure 8:**
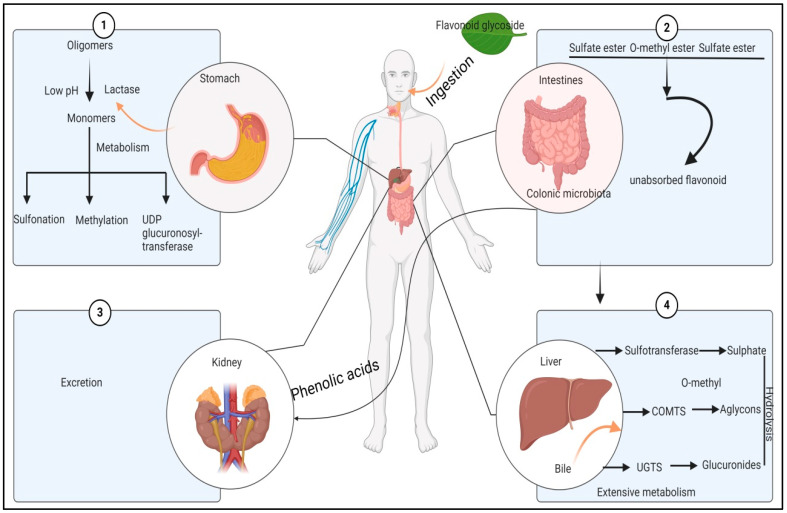
Potential mechanism of antidiabetic activity of mulberry leaves. The numbers indicate action of the mulberry flavonoids on four different parts of the body.

Some past scientific research has shown that there are outstanding hypoglycemic effects of the total flavonoids in mulberry leaves. Mulberry leaves contain several immuinosugars including 1-deoxynojirimycin that can inhibit α-amylase and α-galactosidases [106]. Therefore, the derivatives of nojirimycin are the most effective among all the iminosugars. One oral antidiabetic drug currently marketed in Europe is synthesized from 1-Deoxynojirimycin. The flavonoids and their related constituents present in mulberry leaves also possess antidiabetic effects, as illustrated in Figure 8 The mulberry leaves of the South American mulberry species were found to contain some benzofurane derivatives and are active on STZ-induced diabetic rats [107]. Some of the purified flavone fractions of mulberry were found to activate α-glucosidase enzymes, which in turn increase the blood glucose level [108]. The polysaccharides present in the leaves of mulberry have strong potential for the inhibition of α-glucosidase.

### 5.4. Antimicrobial Activities of Mulberry Leaves

Hitherto, several reports have elucidated the antimicrobial effects of mulberry extracts. Antimicrobials help in the protection against pathogenic microorganisms including bacteria, fungi and viruses. The antimicrobial effects of mulberry include inhibition of microbes and augmentation of antibiotics production. Apart from possessing germicidal effects, mulberry extracts are considered safe for autochthonous intestinal flora. The antiviral activity of mulberry demonstrates its potential as both a promising fruit and a therapeutic drug [109]. Extracts from mulberry leaves contain a variety of beneficial phytoconstituents that can effectively combat harmful pathogens. Mulberry leaves contain Kuwanon C, Mulberrofuran G and albanol B with strong antibacterial activity, with minimum inhibitory concentrations (MICs) ranging from 5 to 30 mg/mL [110]. Zafar et al. (2013) observed that the methanolic extract of *M. alba*, containing Kuwanon G, exhibited strong inhibition of *Streptococcus mutans*, a pathogen responsible for tooth decay [111]. The MIC was determined to be 8.0 mg/mL. The mulberry leaves extract in ethanol shows moderate antibacterial activity, which could be attributed primarily to its flavonoid and phenolic contents, as revealed by structure–activity relationship studies [2]. Some microbes like *E. coli*, *Salmonella typhimurium*, *Staphylococcus epidermis*, *S. aureus*, *Candida albicans* and *Saccharomyces cerevisiae* can be inhibited by the flavonoids present in mulberry leaves [112]. Owing to their wide range of defensive mechanisms, polyphenols were recently suggested to act as potential agents in combating SARS-coronavirus-2 [2]. Utomo et al. (2020) suggested that polyphenols found in grapes, green tea, berries, citrus and curcumin may have potential in combating coronavirus infections [113]. Correspondingly, recent research (Chojnacka et al., 2021), has found that polyphenols have a strong binding ability to the S protein of the viruses including SARS-coronavirus-2, effectively blocking its entry into human cells [114]. In addition, many compounds of *M. alba* exhibit anti-inflammatory effects on the respiratory system. However, these constituents are primarily evaluated in mice in vivo, with inflammation being predominantly caused by LPS [115]. Nonetheless, the effectiveness of polyphenols against coronavirus infection is quite complex and yet to be understood thoroughly.

### 5.5. Cardiovascular Activity

In eastern countries like China, Japan, India,, etc. mulberry extracts are used for treatment of cardiovascular diseases. In China, people use mulberry for reducing blood pressure levels, which is a cause of the cardiovascular disease. Mulberry leaves also assist in lowering serum cholesterol and blood pressure and stopping formation of artherosclerosis [116]. High concentrations of iron present in mulberry leaves help in the better distribution of oxygen, while boosting the production of RBCs. Resveratrol is a flavonoid present in mulberry leaves that augments the formation of nitric oxide (NO), a well-known vasodilator. Nitric oxide makes blood vessels dilate and avoids the formation of blood clots, hence reducing the risk of heart diseases and stroke. Likewise, resveratrol reduces the construction of blood vessels, leading to a decreased probability of heart failure [117]. The administration of *M. alba* has shown promising results in mitigating the myocardial damage caused by isoproterenol in rats [118]. The treated rats exhibited reduced regions of myocarditis and myocardial necrosis, along with decreased levels of cardiac markers. In another study, using a myosin-induced myocarditis model, *M. alba* extracts retained cardiac tissues and prevented excessive inflammation and fibrous tissue buildup. This led to improvements in both systolic and diastolic function of the heart muscle [57]. Experimental mice treated with *M. alba* extracts normalized the heart rates and systolic and diastolic blood pressure. After prolonged use of mulberry leaves, the normal levels of blood vessel activity were restored. This included the reversal of diminished dilatation and increased constriction. Through extensive therapy with *M. alba* leaves, vascular reactivity that was previously impaired, including reduced dilation and heightened contraction, was restored to its normal levels. In vascular smooth muscle cells, activation of the sarcoplasmic reticulum ryanodine receptor mediated contraction, while voltage-gated and receptor-dependent Ca^2+^ channel blockage caused relaxation [119]. According to Yang et al. (2011b), the treatment of *M. alba* leaf extracts also restores the normal levels of circulatory dysfunction markers such as fibrinogen, cell adhesion 1 and NO [120].

### 5.6. Neuroprotective Activity

In cognitive disorders and many other neuronal dysfunctions, medicinal plants are widely used and among them, mulberry is very famous. As previously stated, phenolic compounds, anthocyanins and flavonoids, were identified to have neuroprotective properties [121,122]. The polyphenols and alkaloids that are present in mulberry leaves enhance the cognition and also slow down the process of neuro-degeneration [123]. It has been found that the substances extracted from mulberry leaves can be employed in neurogenesis for neurodegenerative diseases, including t Alzheimer’s disease. They prevent the aggregation of amyloid β-peptide (91-42) and reduce the neurotoxic effects attributable to amyloid β-peptide (1-42) [124]. According to Shahana and Nikalje (2019), the oxyreserveratrol molecule predominant in *M. alba* leaves shows neuroprotective effects in both in vivo and in vitro models of cortical neuronal cells and SHSY5Y cells [125]. As a bioactive chemical, oxyresveratrol shows neuroprotective effects against Alzheimer’s disease. The methanolic extracts of *M. alba* also exhibit anti-dopaminergic effects via inhibition of D2 receptors. Furthermore, in rats, it has been demonstrated that ethanolic extracts of mulberry enhance water maze performance and reduce acetylcholinesterase activity, while increasing neuron density in the hippocampus [2,126,127].

**Table 2:** An overview of the different activities of the bioactive compounds extracted from the leaves of the mulberry.

Type of Activity	Method of Extraction	Activity Unit	Model Cell/Animal Used	Bioactive Compound	Reference(s)
Antioxidant activity	Ethyl acetate	DPPF radical scavenging and reducing activity	Ferric reducing power	Maclurin, rutin, isoquercitin, resveratrol and morin	[128]
Antihyperlipidemic activity	Ethanol	ED_50_	Rats	Mulberroside A (MUL)	[129,130]
Antimicrobial activity	Ethanol	MIC Viable Cell Count	Pathogenic bacteria: *P.aeruginosa*, *E. coli*, *B. subtilis*, *S. mutans*, *S. sanguis*, *S. sobrinus*	Kunwanon G	[131]
Neuroprotective activity	Ethanol	MTT assay	Foot shock induced aggression Water maze test	Isobavachalcone, morachalcone B, moracin N and morachalcone A	[132,133]
Anticancer activity	Ethyl acetate	IC_50_	Hepatocellular carcinoma cells, hepatoma cells	Morushalunin, chalcomoracin guangsangon E, and kuwanon J	[88,100,134]
Antidiabetic activity	Ethanol	HbA1c levels	Rats	sanggenon C, morin, Kuwanon G, morusin, kaempferol, rutin, quercetin, isoquercitrin, 1-deoxynojirimycin	[135]
Anti-obesity activity	Water	Melanin-concentrating hormone receptor subtype 1 (MCH1)	Mice	Chlorogenic acid and Quercitrin	[136]
Tyrosinase inhibitory activity/skin whitening activity	Methanol	SOD	Melanin formation in melan A cells	Mulberroside F	[137]

ED_50_: effective dose 50; carbon tetrachloride; MIC: minimum inhibitory concentration; reactive oxygen species; IC_50_: inhibitory concentration; DPPF: 1,1′-Bis(diphenylphosphino)ferrocene; SOD: superoxide dismutase.

## 6. Methods for Extraction of Bioactive Compounds from Mulberry Leaves

**Figure 9:**
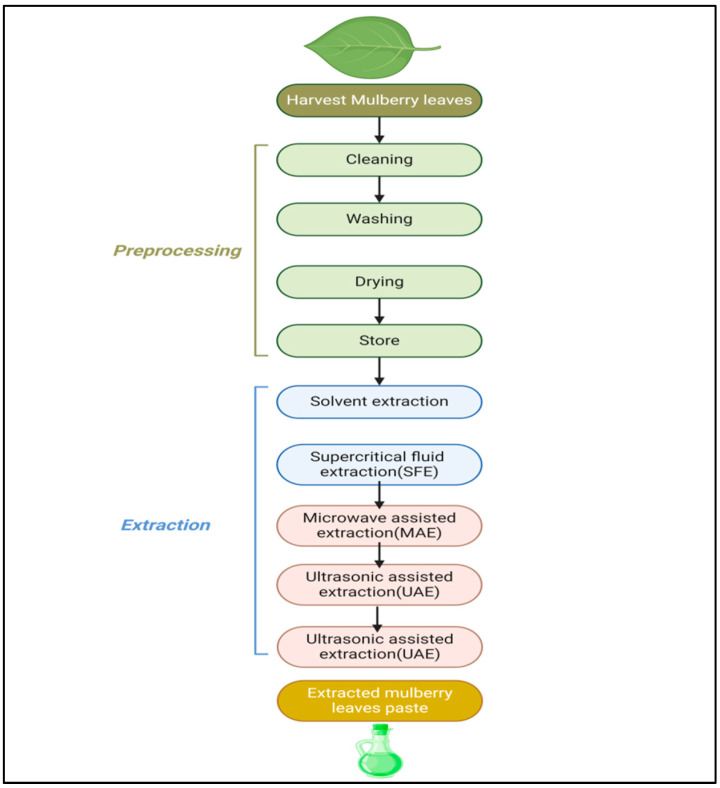
Flow chart showing method of extraction of mulberry leaves.

The method of extraction of mulberry leaves is shown in Figure 9. Extraction techniques for mulberry leaves include solvent extraction, Soxhlet extraction, supercritical fluids, microwave-assisted extraction, ultrasound extraction and pressurized-liquid and solid–liquid extraction. Solvent extraction involves soaking dried mulberry leaves in ethanol or methanol in order to dissolve the bioactive substances. Soxhlet extraction consists of solvent extraction and distillation as a continuous method of extraction. The solvents used in supercritical fluid extraction (SFE) are CO_2_-based supercritical fluids which are compressed under high temperatures and pressure [138]. Microwave-assisted extraction (MAE) turns microwave energy into a heating agent, which helps in accelerating the extraction in the solvent [139]. Ultrasonic-assisted extraction (UAE) generates ultrasound waves which penetrate into plant cells and break barriers, thus facilitating the release of compound. In pressurized-liquid extraction (PLE), temperature and pressure are augmented with the goal of achieving potential separation in a short period of time. The powder of mulberry leaves is obtained by grinding and the extract is essentially prepared by using the solvent [140].

## 7. Toxicological Impacts

The oral toxicity of *Morus alba* ethanol extract at a dose of 300 mg/kg is not fatal and it does not cause histological abnormalities in the liver, spleen or kidneys of mice, only causing a decrease in white blood cell count [141]. The in vivo genotoxicity and acute toxicity of ethanol extracts from *Morus alba* leaves were examined in mice at dosages of 300 and 2000 mg/kg body weight for 14 days ip. There were no fatalities or behavioral problems in the treated mice in the toxicity investigation when compared to the dosage controls. Biochemical, hematological and histological analyses, on the other hand, revealed that intraperitoneal injection generated pathological alterations. The extract did not cause genotoxicity when taken orally [141]. For 14 days, mice were tested for acute toxicity of *Morus alba* ethanol extract at 300 and 2000 mg/kg BW ip. Toxicity was determined by counting the number of micronucleated polychromic red blood cells in the blood of mice administered 75, 150, and 300 mg/kg BW orally. The mice did not die or change behavior when compared to any dose of control. However, after intraperitoneal injection, the extract caused hematological, biochemical and histological changes. At all doses tested by negative control, oral administration of the extract did not cause genotoxicity or significant leukocyte migration (58, 65, 6% inhibition) [142]. The acute and chronic toxicity of *Morus alba* root bark extract was investigated in mice. The extract was administered subcutaneously at a dose of 50–200 mg/kg/day for 3 weeks and 3 months in subacute and chronic toxicity examinations. The extract had no adverse effects on the animal [143]. The toxicity of UP1306, a standardized mixture of *Morus alba* root bark extract and acacia catechu, utilized as a commercial dietary supplement for joint care (500, 1000, 2000 mg/kg, 28 days orally), has been investigated. There was no evidence of morbidity or mortality. In terms of body weight, food intake, hematological, clinical chemistry, organ weight, gross pathology and histology, there were no significant changes between the groups [141]. *Morus alba* leaf ingestion by humans and animals around the world has a long history, implying that the leaves and extracts are largely harmless.

## 8. Conclusions

White mulberry (*Morus alba*) is a remarkable plant that has garnered significant attention due to its rich phytochemical profile and diverse therapeutic potential. This review has provided a comprehensive overview of the key bioactive compounds found in white mulberry, their biosynthesis and the extensive pharmacological activities associated with them. Notably, *M. alba* is recognized for its nutritional richness and antioxidant contents. A synthesis of the recent research indicates that mulberry extracts have been applied for the treatment of diverse ailments, validating its therapeutic properties. Traditional applications of *M. alba* encompass the treatment of diseases like asthma, fever and diarrhea, in addition to its historical use in treating anemia and jaundice. The pharmacological activities of mulberry extracts encompass a multitude of activities including anti-inflammatory, antidiabetic, anticancer, antimicrobial, and neuroprotective effects. These pharmacological properties are attributed to its rich abundance of bioactive compounds such as flavonoids, alkaloids, polysaccharide, polyphenols,, etc. This review has highlighted the abundant presence of flavonoids, especially the potent antioxidant compound rutin, in various parts of the white mulberry plant. These bioactive compounds have demonstrated a wide range of beneficial effects, including antioxidant, anti-inflammatory, antidiabetic, neuroprotective and cardiovascular protective properties. The elucidation of biosynthetic pathways and the genetic regulation of these bioactive compounds in white mulberry has offered valuable insights into the plant’s metabolic mechanisms. This knowledge can inform future efforts to optimize the production of these valuable phytochemicals through targeted breeding, genetic engineering or advanced cultivation techniques. However, this review has also identified several challenges and opportunities for further research. Exploring the precise mechanisms of action underlying the diverse pharmacological activities, investigating the synergistic effects of bioactive compound combinations and developing innovative extraction and formulation strategies remain important areas for future investigation. Lastly, the ethnomedicinal features of *M. alba* warrant further investigation by employing advanced and sophisticated technologies to unravel its hitherto underexplored therapeutic applications for treatment of various diseases in the future.

## 9. Challenges

There are a number of challenges associated with the biosynthesis and pharmacological activities of bioactive compounds of white mulberry (*Morus alba*). These include conducting in-depth mechanistic studies to fully elucidate the molecular pathways and signaling cascades responsible for the diverse pharmacological activities of white mulberry’s bioactive compounds; investigating the specific target receptors, enzymes and transcription factors modulated by these bioactive compounds; exploring the potential synergistic interactions between the various bioactive compounds found in white mulberry, such as flavonoids, phenolic acids and other phytochemicals; assessing the combined therapeutic efficacy of bioactive compound mixtures compared to individual compounds; optimizing the extraction and purification techniques to improve the yield, purity and stability of the bioactive compounds from white mulberry; exploring novel formulation approaches, such as nanoparticles, liposomes or microencapsulation, to enhance the bioavailability and targeted delivery of white mulberry-derived compounds; designing and executing well-designed, large-scale clinical trials to validate the therapeutic efficacy of white mulberry-derived bioactive compounds in various disease models; evaluating the safety, tolerability and optimal dosing regimens for the clinical application of these compounds; investigating the potential of white mulberry-derived bioactive compounds for development as nutraceutical or dietary supplement products; and assessing the feasibility of incorporating these compounds into pharmaceutical formulations for the treatment of various health conditions. The resolution of these challenges can be achieved through the efficient engineering of flavonoid pathways in plants. The vast biodiversity of plants that has emerged over the course of evolution has given rise to a diverse array of flavonoid structures, many of which remain undiscovered. Continued analysis of various plant species holds the potential to unveil novel structures and potentially unexplored metabolic pathways. Future investigations in this field will not only expand our knowledge but also contribute to advancements in floricultural, food, pharmaceutical and chemical industries. Furthermore, as evidence of the beneficial functions of flavonoids in human health continues to accumulate, and the global interest in the use of natural compounds for the prevention and treatment of various pathologies grows, researchers will increasingly focus on furthering our understanding of flavonoids. This trend is anticipated to persist and gain momentum in the years to come.

## Figures and Tables

**Table 1:** A list of the flavonoid compounds extracted from mulberry plants using different methods.

Sr. No.	Compound	Method of Extraction	Chemical Structure	Bioactive Potential	Reference(s)
1	Rutin	Methanol extraction	C_27_H_30_O_16_	Inhibition of peroxidation and acts as an antioxidant, reverts β-amyloid toxicity	[33]
2	Isorhamnetin	Ethanol extraction	C_16_H_12_O_7_	Antioxidant (IC50-14.3 µM), anti-inflammatory (IC50-3.9 µM), Neuroprotective, Anticancer	[34,35]
3	Myricetin	Methanol extraction	C_15_H_10_O_8_	Antioxidant (IC50-3.1 µM), anti-inflammatory (IC50-5.7 µM), anti-cardiovascular	[32,36,37]
4	Quercetin	Solvent Extraction	C_15_H_10_O_7_	Antioxidant	[38,39]
5	Kaempferol	Solvent Extraction	C_15_H_10_O_6_	Ameliorate hyperglycemia, antioxidant effect	[40]
6	Quercetin-3,7-di-O-β-D-glucopyranoside	Solvent Extraction	C_27_H_30_O_17_	Antioxidant (IC50-12.8 µM), anti-inflammatory (IC50-9.3 µM)	[41,42]
7	Kaempferol-3,7-di-O-β-glucopyranoside	Solvent Extraction	C_27_H_30_O_16_	Antioxidant (IC50-16.4 µM), anti-inflammatory (IC50-12.1 µM)	[41,42]
8	Quercetin-3-O-β-D-glucopyranoside (isoquercitrin)	Solvent Extraction	C_21_H_20_O_12_	Reduce oxidative stress	[43,44]
9	Kaempferol-3-O-β-D-glucopyranoside (milk vetch glycoside)	Solvent Extraction	C_21_H_19_O_11_	Antioxidant (IC50-20.3 µM), anti-inflammatory (IC50-14.2 µM)	[43,44]
10	Quercetin-3-O-α-L-rhamnosyl-(1-6)-β-glucopyranose (rutin)	Solvent Extraction	C_27_H30O_16_	Antioxidant (IC50-9.7 µM), anti-inflammatory (IC50-7.4 µM), Neuroprotective activity	[44,45]
11	Kaempferol-7-O-β-D-glucopyranoside	Solvent Extraction and Chromatography technique	C_21_H_20_O_11_	Antioxidant (IC50-17.9 µM), anti-inflammatory (IC50-11.6 µM)	[46]
12	Quercetin-3-O-(6″-O-acetyl)-β-D-glucopyranoside	LC-MS	C_23_H_22_O_13_	Antioxidant (IC50-14.2 µM), anti-inflammatory (IC50-9.8 µM)	[26]
13	Kaempferol-3-O-α-L-rhamnosyl-(1-6)-β-glucopyranoside	HPLC	C_33_H_40_O_20_	Antioxidant (IC50-16.7 µM), anti-inflammatory (IC50-10.9 µM)	[41]
14	Quercetin-3-O-β-D-glucosyl-(1-6)-β-glucopyranoside	HPLC	C_21_H_20_O_12_	Antioxidant (IC50-11.4 µM), anti-inflammatory (IC50-8.2 µM)	[41,42]
15	Kaempferol-3-O-(6″-O-acetyl)-β-D-glucopyranoside	HPLC	C_23_H_22_O_12_	Antioxidant (IC50-15.6 µM), anti-inflammatory (IC50-10.3 µM)	[41,42]
16	Quercetin-3-O-(6″-O-malonyl)-β-D-glucopyranoside	HPLC	C_24_H_22_O_15_	Antioxidant (IC50-12.9 µM), anti-inflammatory (IC50-9.1 µM)	[41,42]
17	Kaempferol-3-O-(6″-O-malonyl)-β-D-glucopyranoside	HPLC	C_24_H_22_O_14_	Antioxidant (IC50-14.8 µM), anti-inflammatory (IC50-10.6 µM)	[41,42]
18	Kaempferol-3-O-β-D-glucosyl-(1-6)-β-glucopyranoside	Chromatography technique	C_21_H_20_O_11_	Antioxidant (IC50-17.2 µM), anti-inflammatory (IC50-11.4 µM)	[46]
19	Quercetin-3-O-α-L-rhamnopyranoside	LC-MS	C_21_H_20_O_11_	Antioxidant (IC50-10.2 µM), anti-inflammatory (IC50-7.8 µM)	[45]
	Kaempferol-3- O-α-L-rhamnoside	Liquid Chromatography technique	C_21_H_20_O_10_	Antioxidant (IC50-14.3 µM), anti-inflammatory (IC50-9.7 µM)	[47]
	Quercetin-3-O-rhamnose-7-O-glucoside	Chromatography technique	C_27_H_30_O_16_	Antioxidant (IC50-12.0 µM), anti-inflammator (IC50-8.6 µM)	[48]
	Quercetin-3-O-glucose-7-O-rhamnoside	Chromatography technique	C_27_H_30_O_16_	Antioxidant (IC50-11.8 µM), anti-inflammatory (IC50-8.4 µM)	[48]

HPLC: High-performance liquid chromatography; LC-MS: Liquid chromatography–mass spectrometry.
